# Contact Lens Health Week — August 19–23, 2019

**DOI:** 10.15585/mmwr.mm6832a1

**Published:** 2019-08-16

August 19–23, 2019, marks the sixth annual Contact Lens Health Week. In collaboration with partners from clinical, public health, industry, and regulatory sectors, CDC is promoting healthy contact lens wear and care practices to reduce the risk for eye infections among the approximately 45 million persons in the United States who wear contact lenses. Studies conducted following outbreaks of rare but serious eye infections in the United States have found that these infections occur most frequently in contact lens wearers who do not take proper care of their contact lenses, indicating a need to promote safer wear and care (*1*).

A report in this issue of *MMWR* reviews reported provision and receipt of contact lens wear and care recommendations among providers and patients in the United States (*2*). One third of lens wearers recalled never hearing any lens care recommendations. Most eye care providers reported sharing recommendations always or most of the time. Developing effective health communication messages can help eye care providers communicate with their patients. Practicing proper contact lens hygiene and regularly visiting an eye care provider are important actions for keeping contact lens wearers' eyes healthy.

Additional information on Contact Lens Health Week and the proper wear and care of contact lenses is available at https://www.cdc.gov/contactlenses.
